# Automated macrophage counting in DLBCL tissue samples: a ROF filter based approach

**DOI:** 10.1186/s12575-019-0098-9

**Published:** 2019-07-01

**Authors:** Marcus Wagner, René Hänsel, Sarah Reinke, Julia Richter, Michael Altenbuchinger, Ulf-Dietrich Braumann, Rainer Spang, Markus Löffler, Wolfram Klapper

**Affiliations:** 1 Institute for Medical Informatics, Statistics and Epidemiology (IMISE), University of Leipzig, Härtelstr. 16–18, Leipzig, 04107 Germany; 2 Department of Pathology, Hematopathology Section and Lymph Node Registry, University of Kiel/University Hospital Schleswig-Holstein, Arnold-Heller-Str. 3, Haus 14, Kiel, 24105 Germany; 3 Institute of Functional Genomics, Statistical Bioinformatics, University of Regensburg, Am BioPark 9, Regensburg, 93053 Germany; 4 Faculty of Electrical Engineering and Information Technology, Leipzig University of Applied Sciences (HTWK), P. O. B. 30 11 66, Leipzig, 04251 Germany; 5 Fraunhofer Institute for Cell Therapy and Immunology (IZI), Perlickstr. 1, Leipzig, 04103 Germany

**Keywords:** Macrophage, Immunohistochemical staining, CD14, CD163, Automated cell counting, ROF filtering, Floating threshold, Rule-based detection

## Abstract

**Background:**

For analysis of the tumor microenvironment in diffuse large B-cell lymphoma (DLBCL) tissue samples, it is desirable to obtain information about counts and distribution of different macrophage subtypes. Until now, macrophage counts are mostly inferred from gene expression analysis of whole tissue sections, providing only indirect information. Direct analysis of immunohistochemically (IHC) fluorescence stained tissue samples is confronted with several difficulties, e.g. high variability of shape and size of target macrophages and strongly inhomogeneous intensity of staining. Consequently, application of commercial software is largely restricted to very rough analysis modes, and most macrophage counts are still obtained by manual counting in microarrays or high power fields, thus failing to represent the heterogeneity of tumor microenvironment adequately.

**Methods:**

We describe a Rudin-Osher-Fatemi (ROF) filter based segmentation approach for whole tissue samples, combining floating intensity thresholding and rule-based feature detection. Method is validated against manual counts and compared with two commercial software kits (Tissue Studio 64, Definiens AG, and Halo, Indica Labs) and a straightforward machine-learning approach in a set of 50 test images. Further, the novel method and both commercial packages are applied to a set of 44 whole tissue sections. Outputs are compared with gene expression data available for the same tissue samples. Finally, the ROF based method is applied to 44 expert-specified tumor subregions for testing selection and subsampling strategies.

**Results:**

Among all tested methods, the novel approach is best correlated with manual count (0.9297). Automated detection of evaluation subregions proved to be fully reliable. Comparison with gene expression data obtained for the same tissue samples reveals only moderate to low correlation levels. Subsampling within tumor subregions is possible with results almost identical to full sampling. Mean macrophage size in tumor subregions is 152.5±111.3 *μ*m^2^.

**Conclusions:**

ROF based approach is successfully applied to detection of IHC stained macrophages in DLBCL tissue samples. The method competes well with existing commercial software kits. In difference to them, it is fully automated, externally repeatable, independent on training data and completely documented. Comparison with gene expression data indicates that image morphometry constitutes an independent source of information about antibody-polarized macrophage occurence and distribution.

**Electronic supplementary material:**

The online version of this article (10.1186/s12575-019-0098-9) contains supplementary material, which is available to authorized users.

## Background

Diffuse large B-cell lymphoma (DLBCL), the most frequent mature aggressive B-cell lymphoma in adults, is characterized by very heterogeneous pathological, clinical, and biological features [1]. Additionally to the neoplastic B-cells, cancerous tissue contains high numbers of various subsets of T-cells, macrophages, mast cells and stromal cells [1, 2]. The composition of this tumor microenvironment has attracted considerable interest since it turned out to affect the clinical outcome. Besides of overall histological inspection, it has been largely investigated by molecular procedures as gene expression profiling (GEP) [3, 4] as well as by morphometric image analysis [5, 6]. Based on GEP results, two biologically and clinically distinct molecular subtypes of DLBCL were identified, namely activated B-cell-like subtype (ABC) and germinal center B-cell-like subtype (GCB) [7, 8], the latter being associated with a favorable prognosis. Prognostic effects by different signatures of the tumor microenvironment were also found by Lenz et al. [9]. In particular, a signature associated with increased overall survival included components of the extracellular matrix and genes that are characteristically expressed in cells from the monocytic lineage.

An important component of tumor microenvironment are infiltrating tumor-associated macrophages (TAMs). As yet, the role of TAMs and their possible importance for prognosis is a controversially discussed item. Although TAMs have been associated with immunomodulation in other tumor entities [10, 11], their functional role in the DLBCL tumor microenvironment is still not fully defined [12–15]. A typical marker used for its identification is CD163. In the present study, besides of CD163, we use CD14 as a further specific marker for monocytes and macrophages. The choice of this particular marker pair has been motivated by the intention of future testing whether the ratio of CD14/CD163 could be used as a prognostic factor for clinical outcome in DLBCL patients.

Until now, macrophage counts are either inferred from GEP analysis of whole tissue sections or by manual counting in immunohistochemically (IHC) fluorescence stained tissue microarrays (TMA) or high-power fields (HPF) [16, 17]. However, due to the heterogeneity of the tumor microenvironment, counts within TMAs and HPFs cannot be considered as representative. Consequently, morphometric image analysis and related macrophage counting should be performed for *whole IHC stained tissue slides* instead of for small subareals.

For several reasons, *fully automated counting of IHC stained macrophages* within tissue sections is still a difficult task [18–20]. First, the size and shape of the macrophages are highly variable, thus largely impeding a recognition by prior shape information. Second, the intensity of the staining shows a large variation as well, even within a single tissue sample or for different parts of a single macrophage. Third, we must deal with cropped or squeezed cells as well as with macrophages located outside the focal plane, appearing as defocused features within the images. Further, as far as fluorescent-labeled antibodies are used, we must cope with autofluorescence of other structures, e.g. erythrocytes, in the tissue. For these reasons, the most popular strategies for cell segmentation [21], i.e. (fixed or adaptive) intensity thresholding and elementary feature detection, as implemented in most commercial software kits, will be confronted with serious difficulties when applied to macrophage segmentation.

In the present study, therefore, we describe a novel *ROF filter based segmentation approach*, which allows for fully automated macrophage counting in whole tissue sections, and avoids the above mentioned difficulties, at least in part. More precisely, we will combine a strategy of floating intensity thresholding with a rule-based feature detection in single-channel images. The latter has been suggested e.g. in Steiner et al. [22] for detection of IHC stained leukocytes. Our method is deterministic, fully automated, externally repeatable (no dependence on training data) and — in difference to most commercial software packages — completely documented. It will be validated against manual macrophage counts in a set of 50 test images.

Further, *our novel method will be compared with different existing segmentation approaches*. For the mentioned test image set, we perform a comparison with the output of two commercial cell segmentation software kits (Tissue Studio 64, Definiens AG, Munich, Germany, and Halo, Indica Labs, Corrales, New Mexico, USA) as well as with a straightforward machine-learning approach (training and application of a region-based convolutional neural network). Next, our method and both commercial packages will be applied to a set of 44 whole tissue sections, and outputs will be compared with each other as well as with GEP data available for the same tissue samples. In a final step, the ROF based segmentation approach will be applied to 44 expert-specified tumor subregions for testing selection and subsampling strategies. To the best of the authors’ knowledge, a comparative analysis of automated macrophage segmentation approaches is being conducted for the first time.

## Methods

### Preparation and staining of tissue samples

44 biopsy specimens of DLBCL were selected from the files of the Lymph Node Registry Kiel based on availability of material. Core needle biopsies were excluded. Formalin-fixed paraffin-embedded (FFPE) tissue was sliced into 2 *μ*m thin slides and, additionally to a conventional HE-staining, an immunohistochemical staining was done with antibodies against CD14 (Clone EPR3653; Cell Marque, Rocklin, CA, USA; 1:10) and CD163 (Clone 10D6; Novocastra, Leica Biosystems, Wetzlar, Germany; 1:100). Briefly, after deparaffinization in xylene and rehydration in alcohol, tissue sections were incubated for 3 min in citrate buffer (pH 6) within a pressure cooker. The slides were washed in PBS and then incubated for 1 h with a mixture of the primary antibodies in antibody-diluent (medac GmbH, Wedel, Germany). After incubation with the primary antibodies, the sections were washed in PBS and then incubated with a mixture of the secondary fluorescent-labeled antibodies in PBS for 1 h. As secondary antibodies, donkey anti rabbit Alexa 488 and donkey anti mouse Alexa 555 were used (both from Invitrogen, Thermo Fisher Scientific, Waltham, MA, USA; 1:100). After washing in PBS the slices were incubated with DAPI (Invitrogen, Thermo Fisher Scientific, Waltham, MA, USA; 1:5000) for 2 min, washed in PBS and cover-slipped with mounting medium. Use of tissue was in accordance with the guidelines of the internal review board of the Medical Faculty of the Christian-Albrechts-University Kiel, Germany (No. 447/10).

### Image acquisition, selection of tumor subregions and ROIs

Images were generated by Hamamatsu Nanozoomer 2.0 RS slide scanner (Hamamatsu Photonics, Ammersee, Germany) with 20 × magnification. For every fluorescent immunostained tissue slide, the whole tissue sample as well as a tumor subregion were imaged, resulting in single images for the Alexa 488, Alexa 555, and DAPI channel, respectively, and an overlay picture of the channels. Raw image data were saved in.ndpi format (single-channel images) or.ndpis format (overlay image), respectively. Pixel size is 0.45 *μm* × 0.45 *μ*m in all images.

In order to select a tumor subregion within a whole tissue sample, the tumor area was defined and marked by a pathologist by inspection of the HE-stained slice. Subsequently, within the immunostained slice, a suitable subregion of the tumor area not larger than 10 mm^2^ has been selected depending on tissue and staining quality (no tissue artifacts, no scratches or folding in the tissue, no overstaining) and captured. The position of the selected tumor subregion has been marked within the raw data by use of the software kit NDP.view 2 (Hamamatsu Photonics, Ammersee, Germany), which is available as freeware [23].

**Fig. 1:**
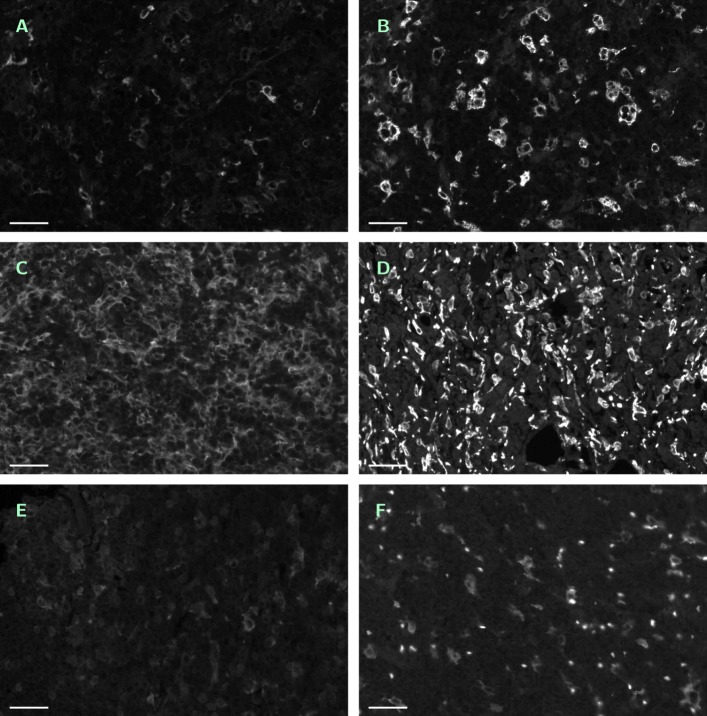
Six typical examples of single-channel ROIs. Contrast enhanced by factor 2 in all images, scale bar 45 *μ*m. **a** — No. 11 (CD14 ^+^/488 nm). **b** — No. 36 (CD163 ^+^/555 nm), same region as in **a**. **c** — No. 01 (CD14 ^+^/488 nm), tightly packed and squeezed macrophages. **d** — No. 28 (CD163 ^+^/555 nm), tightly packed and squeezed macrophages, many erythrocytes. **e** — No. 12 (CD14 ^+^/488 nm), weak contrast. **f** — No. 40 (CD163 ^+^/555 nm), defocused and weakly stained macrophages, strongly autofluorescent erythrocytes

From 25 randomly selected tumor subregions, ROIs of 900 × 600 px (0.109 mm^2^) size for manual counting and comparison of image analysis methods have been singled out (CD14 ^+^/488 nm and CD163 ^+^/555 nm channels). Note that the ROIs have been selected under the viewpoint of reflecting the several difficulties of automated macrophage recognition, see Fig. 1.

In order to prepare the scans for image analysis, raw data were converted into uncompressed .tif format and, in the case of whole tissue samples and tumor subregions, sliced into tiles of 1000 × 1000 px (0.202 mm^2^) size, using the software package ImageJ with the extension ndpitools [24]. Since all obtained images are monochrome, they have been further converted from RGB into greyscale mode using the modulus *I*_*grey*_=| *I*_*RGB*_ | of the RGB vector and finally saved in losslessly compressed.png format. Thus we end up with 50 ROIs, 44 datasets for whole tissue samples and 44 datasets for tumor subregions, each comprising image data at three different immunostainings. Note that the image acquisition as well as the tiling resp. selection of the ROIs has been organized such that no misalignment between the scans at the different wavelengths occurred.

Let us remark that a further staining with Pax5 (polyclonal; Santa Cruz Biotechnology, Heidelberg, Germany; 1:100) and donkey anti goat Alexa 647 (Invitrogen, Thermo Fisher Scientific, Waltham, MA, USA; 1:100) has been simultaneously performed and imaged but all related information, as it is not concerned with macrophages, has been completely excluded from the following analyses.

### Fully automated ROF filter based segmentation

**Fig. 2:**
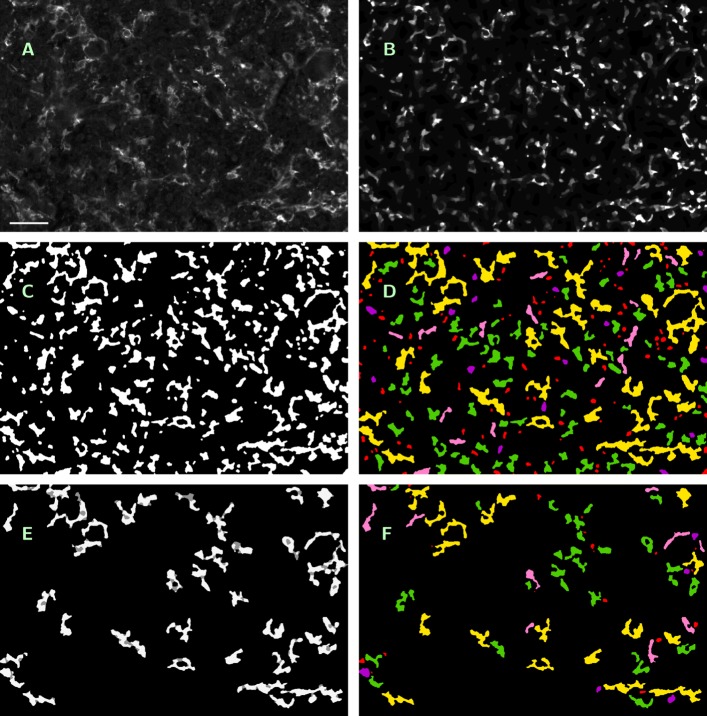
Visualization of processing steps in ROF filter based segmentation. **a** — Original single-channel image (ROI No. 09, CD14 ^+^/488 nm), contrast enhanced by factor 3, scale bar 45 *μ*m. **b** — Cartoon of **a** as result of Steps 1 and 2, contrast enhanced by factor 6. **c** — Features to be examinated in **b** after masking with initial threshold *i*=3 (Steps 4 − 6). **d** — Feature classification in **c** (Steps 7 − 9): saved by Rule 1 for further processing (yellow); excluded by Rule 2 (red), Rule 3a (purple) or Rule 3b (pink); accepted as macrophages (green). Rule 3c caused no exclusions here. **e** — Features to be examinated in **b** after masking with incremented threshold *i*=4 (white); pixels saved in **d** but masked now (grey) (Step 10). **f** — Feature classification in **e**, color encoding as before. Rule 3c caused no exclusions again

*a) Method description.* The described method originates as a substantial further development of the approach presented in Bredies et al. [25], where IHC stained photoreceptor segmentation was performed with data-dependent but fixed intensity thresholding and without application of geometric rules for feature segmentation. Some of the steps described below are visualized in Fig. 2.

After initialization of the parameters *(Step 0)*, subtraction of a median-filtered version *I*^(1)^ from the original image *I*^(0)^*(Step 1)*, which results in a brightness-normalized, unsharply masked image *I*^(2)^= max (*I*^(0)^−*I*^(1)^, 0), we apply the Rudin-Osher-Fatemi (ROF) filter [26] *(Step 2)*, ending up with *I*^(3)^. ROF filtering constitutes a well-established standard procedure in image processing, resulting in a sligthly coarsened, cartoon-like version of the input image which, nevertheless, conserves the original edge structure. The procedure allows for a surprisingly efficient numerical realization [27], pp. 175 ff. Steps 0 − 2 are analogous to the algorithm described in Bredies et al. [25]. We refer to the appendix of this paper for an outline of the mathematical background of the ROF approach.

Next, we extract the evaluation subregion to which the macrophage segmentation has to be applied (i.e., the part of the image where tissue is present). For this purpose, we apply Steps 1 and 2 to the DAPI image, which is available together with *I*^(0)^. From the obtained DAPI cartoon, we generate a black-and-white mask *I*_*eval*_ by masking all pixels with intensity less than 10 at 8bit scale with black and covering every remaining pixel with a white 31 × 31 px square centered at the given position *(Step 3)*. In the case of application of the method to the ROIs, this step is being skipped, and the evaluation subregion is assumed to coincide with the ROI image as a whole. Note that, in difference to the following step, the application of a fixed threshold is possible due to the much more regular structure of the DAPI image. The threshold value has been experimentally chosen.

In difference to [25], the cartoon *I*^(3)^ will be segmented with a floating intensity threshold instead of a fixed one, and features will be identified as macrophages by application of a set of several geometrical rules. This subprocedure, which has been newly developed, will be described in more detail. For the geometrical description of a feature *F*, we employ the following variables: the size *s*(*F*) of the feature itself, the size *c*(*F*) of the convex hull of the feature, the ratio *r*(*F*) of the principal axes’ lengths of the smallest ellipse covering the feature, the perimeter *p*_1_(*F*) of the feature and the perimeter *p*_2_(*F*) of a circle with equal area to the feature *F*. Further, we define the parameters *s*_*min*_ and *s*_*max*_ — minimal and maximal feature size (in px), *c*_*min*_ — minimal area excess of the convex hull (in percent), *r*_*max*_ — maximal ratio of axes, and *p*_*max*_ — maximal excess of the feature perimeter *p*_1_ when compared with the perimeter of a circle with equal area *p*_2_.

We start at the intensity threshold *i*, which will be given as the mean intensity of *I*^(3)^, rounded to the next integer value, and the feature mask *I*^(3)^(*i*):=*I*^(3)^. Using *I*_*eval*_, we mask in *I*^(3)^(*i*) all pixels outside the obtained evaluation subregion *(Step 4)*. Now we perform the first segmentation step by masking in *I*^(3)^(*i*) all pixels with intensity less than *i*, subsequent labeling *(Step 5)* and inspecting the connected features *F*_*j*_,*j*=1,..., *N*(*i*), in *I*^(3)^(*i*)*(Step 6)*. Each of the features *F*_*j*_ will be classified by the following rules.

1) If *s*_*max*_<*s*(*F*_*j*_) then do nothing, reserving the too large feature for further analysis with incremented intensity threshold *(Step 7)*. 2) If *s*(*F*_*j*_)<*s*_*min*_ then neglect the feature as too small and mask it in *I*^(3)^(*i*)*(Step 8)*. 3) If *s*_*min*_≤*s*(*F*_*j*_)≤*s*_*max*_ then test whether the feature satisfies all of the following three criteria: 3a) *c*(*F*_*j*_)/*s*(*F*_*j*_)≥1+*c*_*min*_/100 (the feature is not too round), 3b) *r*(*F*_*j*_)≤*r*_*max*_ (the feature is not too elongated), and 3c) *p*_1_(*F*_*j*_)/*p*_2_(*F*_*j*_)≤*p*_*max*_ (the feature’s boundary is regular enough). If yes, save the feature *F*_*j*_ into the output mask *I*_*segm*_, interpreting it as macrophage, and mask it in *I*^(3)^(*i*). If at least one of the three criteria fails then neglect the feature and mask it in *I*^(3)^(*i*) as well *(Steps 9 and 10)*.

**Fig. 3:**
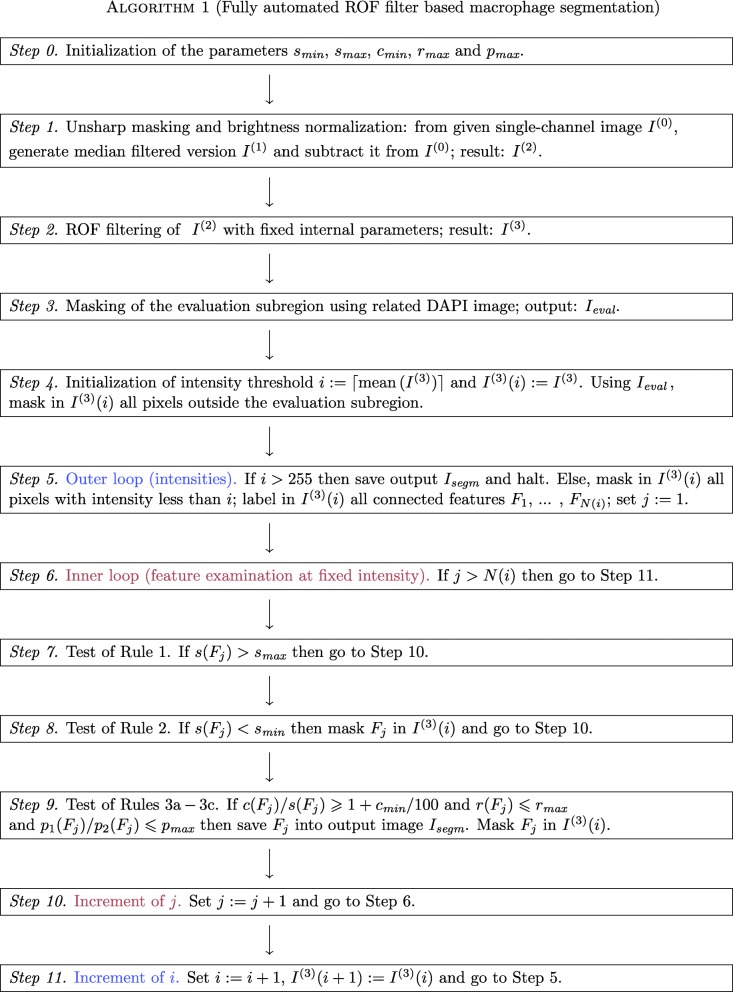
Flowchart of ROF filter based segmentation algorithm

As a result of the classification, we end up with a masked version *I*^(3)^(*i*) of the cartoon and (possibly) a set of features to be interpreted as macrophages, written into the output mask *I*_*segm*_. Now the segmentation step is repeated with incremented intensity threshold *i*=*i*+1, further application of masking to *I*^(3)^(*i*+1):=*I*^(3)^(*i*)*(Step 11)* and geometrical analysis of the remaining features. Thus we repeat subsequent segmentation steps until the maximal intensity is reached. The complete algorithm is summarized in Fig. 3 again.

**Fig. 4:**
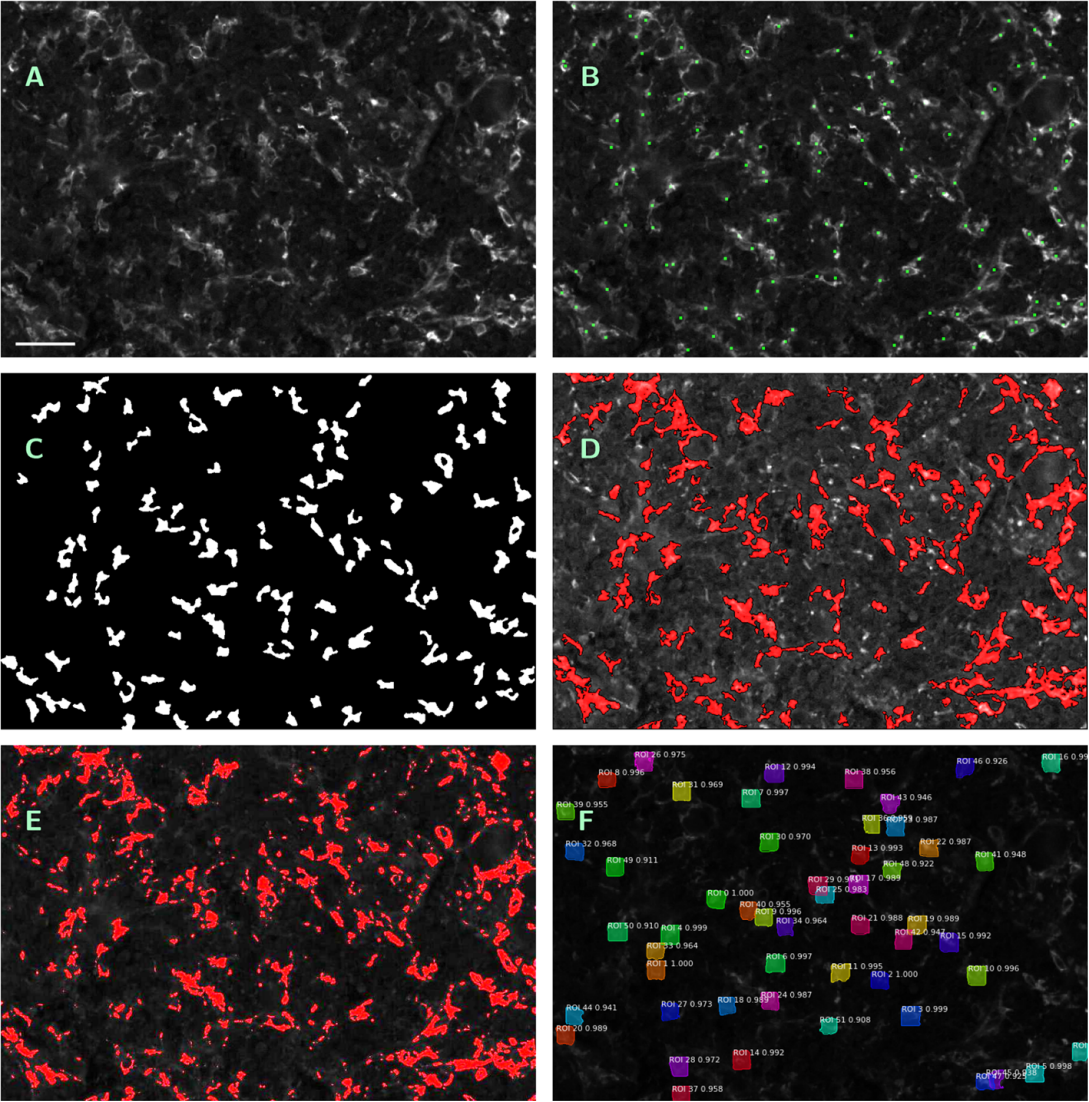
Visualization of outputs of different segmentation methods. **a** — Original single-channel image (ROI No. 09, CD14 ^+^/488 nm), contrast enhanced by factor 3, scale bar 45 *μ*m. **b** — Manual count within **a**; macrophages tagged with green squares. **c** — Output mask *I*_*segm*_ of ROF filter based segmentation (S1), (S2). **d** — Annotated image as output of software kit Tissue Studio (S3), contrast enhanced by factor 6, detected macrophage area in red. **e** — Annotated image as output of software kit Halo (S4), contrast enhanced by factor 3, detected macrophage area in red. **f** — Annotated image as output of machine learning method Mask R-CNN (S5)

*b) Input, output and implementation.* As input for the method, a single-channel greyscale image is required. In the case of whole tissue samples and tumor subregions, the related greyscale DAPI image must be provided as well. The output of the procedure are three black-and-white masks. *I*_*eval*_, the first one, contains the evaluation subregion. Into *I*_*segm*_, all detected macrophages are plotted as white features which are, as a consequence of the organization of the processing steps, mutually disjoint, see Fig. 4c. Into the third mask *I*_*conv*_, we plot all convex hulls conv (*F*) of the detected macrophages *F*. All result images are of the same size as the input image. Further, the method provides the total area of the evaluated subregion marked in *I*_*eval*_, the number of features in *I*_*segm*_ as macrophage count and the total area marked in *I*_*conv*_, i.e. the cumulative area of the convex hulls of the obtained features, as macrophage area. We refer to the obtained count as to method (S1) and to the obtained cumulative area as to method (S2).

The algorithm has been implemented as a series of MATLAB procedures. They have been tested on MATLAB 9.4.0.813654 (R2018a) and require the MATLAB Image Processing Toolbox [28, 29]. For the ROF filtering in Step 2, the numerical method from [30] is applied. The window size for the median filter (31 ×31 px) as well as the internal parameters of the ROF filtering are being fixed from the outset. The geometrical parameters from Steps 7 − 9 must be initialized as well. For the analysis of the ROIs, we used *s*_*min*_=140,*s*_*max*_=800,*c*_*min*_=7.5,*r*_*max*_=3 and *p*_*max*_=2. For the analysis of the whole tissue samples and the tumor subregions, we set the parameters to *s*_*min*_=160,*s*_*max*_=1500,*c*_*min*_=7.5,*r*_*max*_=3 and *p*_*max*_=2.5.

The parameter *s*_*min*_ has been set above 140 px in order to exclude the misidentification of erythrocytes (with a mean diameter of about 6 *μ*m and a corresponding mean area of ca. 100 px) as (parts of) macrophages. The setting of *s*_*max*_ is well in agreement with the mean macrophage area reported in the “Results” section below. The values of the parameters *c*_*min*_,*r*_*max*_ and *p*_*max*_ have been experimentally found. No particular attempts for performance tuning have been made.

Let us remark that dependency on proprietary software can be completely removed, e.g., by reimplementation of the ROF segmentation procedures in the freeware environment OCTAVE [31].

*c) Availability and usage.* We made the MATLAB procedures publicly accessible (CC0 1.0 Universal Public Domain Dedication or GNU General Public License v3) at the Leipzig Health Atlas repository under the address [32]. Execution assumes that a single image set, consisting of three greyscale images representing the CD14 ^+^/488 nm, CD163 ^+^/555 nm and DAPI channels, as well as the procedures are stored in the MATLAB working directory. Output images and logfile will be saved at the same location. To start the analysis, type rof_segm_public_step_00_masterfile, which subsequently calls the other procedures, within the MATLAB command window. You will be asked to enter the image filenames and to confirm the parameter settings. Progress of segmentation can be traced by display messages. Parameters are set by default to the values used for the analysis of the whole tissue samples and the tumor subregions as described in the subsection above. They can be changed within the file rof_segm_public_step_01_parameters.m. Modification of the basic procedure in order to enforce batch processing may be easily effected but is left to the user as it depends strongly on the particular structure of the dataset to be analyzed.

### Other segmentation methods

*a) Commercial software kits.* We applied two commercial software packages to the images. The first one is *Tissue Studio 64, v3.6.1* (Definiens AG, Munich, Germany) [33]. In the case of the ROIs, single-channel images (at 488 and 555 nm) in.png format were separately uploaded and analyzed. Magnification was defined using the image metadata (20 × magnification, pixel resolution 0.45 *μ*m/px), stained area was analyzed in “Marker Area Detection” mode. The minimal feature size was set to 30 *μ*m^2^ in order to exclude fragments of macrophage protrusions from counting. Thresholds for IHC marker intensity staining were manually adapted for each image (within ranges from 10 to 23 for CD14 ^+^/488 nm and from 11 to 26 for CD163 ^+^/555 nm channel on a 8bit scale). For the analysis of the whole tissue samples, .ndpis files were uploaded. In order to define the evaluation subregion, all layers were used for tissue background separation. Instead of using the auto-threshold function of the software kit, homogeneity threshold was set on 0.2, brightness control was manually adapted within a range from 2 to 6, tissue minimum size was set between 10 and 2000 *μ*m^2^ depending on the tissue sample. Areas with overstaining, scratches or folding were excluded by manual marking. Then the CD14 ^+^/488 nm and CD163 ^+^/555 nm channels have been analyzed independently from each other in “Marker Area Detection” mode. Thresholds were manually set in ranges from 13 to 40 for CD14 ^+^/488 nm and from 12 to 45 for CD163 ^+^/555 nm channel on a 8bit scale. As output, the software provides the total area analyzed and the areas bearing the respective stainings. Graphical output is an annotated version of the original image with marking of the detected area, see Fig. 4d. We refer to the me thod as to (S3).

The other software kit is *Halo, v2.1.1637.11* (Indica Labs, Corrales, New Mexico, USA) [34]. Magnification was set to 0.45 *μ*m/px, and “Area Quantification FL v1.2” mode was applied. In the case of the ROIs, single-channel images (at 488 and 555 nm) in.png format were separately uploaded and analyzed. For the analysis of the whole tissue samples, .ndpi files were uploaded. Based on simultaneous inspection of all layers, the evaluation subregion has been marked manually, excluding at the same time areas with apparent overstaining, scratches or folding. Then the CD14 ^+^/488 and CD163 ^+^/555 nm channels have been analyzed independently from each other. Again, thresholds for IHC marker intensity staining were adapted manually for each image (within ranges from 0.1 to 0.16 for CD14 ^+^/488 nm and from 0.125 to 0.19 for CD163 ^+^/555 nm channel for the ROIs and from 0.021 to 0.097 for CD14 ^+^/488 nm and from 0.047 to 0.279 for CD163 ^+^/555 nm channel for the whole tissue samples on a float scale). As output, the software provides the total area analyzed and the stained areas. Graphical output is an annotated version of the original image with marking of the detected area, see Fig. 4e. We refer to the method as to (S4).

*b) Machine learning method (Mask R-CNN).* Mask R-CNN is a region-based convolutional neural network, providing bounding boxes for candidate target objects together with a binary mask for the objects themselves [35]. It depends on two sets of greyscale images annotated with bounding boxes for the contained features of interest, which are used for training and validation, respectively. In our case, the training set was built from 10 randomly selected ROIs (20 % of data available), and the validation set consisted of further 5 randomly selected ROIs (10 % of data available), thus leaving 35 ROIs for the application of the method. Selection and annotation of training resp. validation features within the original images was performed by assigning a centered 31×31 px square subregion around every tag obtained by manual counting (whose output is available as a mask) as a valid training feature. Annotation was performed by software package VGG Image Annotator [36]. Annotated images were converted into backbone feature map of size 32×32×2048 by standard convolutional neural network ResNet-101 [37]. Based on the obtained training data, the remaining 35 ROIs (at 488 and 555 nm, 70 % of data available) were subjected to segmentation with Mask R-CNN, using the implementation available at [38]. Single-channel images were uploaded in .png format. The output of the method is an annotated version of the original image with bounding boxes for the detected macrophages and a black-and-white mask of the same size as the input image, into which all detected macrophages have been plotted, see Fig. 4f. For counting and area evaluation, features of size less than 140 px were ignored. We refer to the obtained count as to method (S5) and to the obtained cumulative area of macrophages, as derived from the black-and-white mask, as to method (S6).

### Mutual comparison of the segmentation methods

*a) Manual count as reference basis.* Within single channel images of the ROIs (at CD14 ^+^/488 nm and CD163 ^+^/555 nm), macrophage cells were marked with a 3×3 px cross and manually counted (see Fig. 4b, wherein, for better visibility, the cross-shaped detection marks have been replaced by squares centered at the same pixel). Tags have been saved into a black-and-white mask of equal size as the original image. We refer to the manual count as to method (MC).

*b) Method comparison by means of the ROIs.* To the ROI image set, segmentation methods (S1) − (S6) have been applied and subsequently compared. For this comparison, the relative error turns out to be an inadequate measure. Indeed, since manual counts range from 8 to 311 macrophages per ROI, the relative error would vary from 0.32 % to 12.5 % per erroneously counted single feature, thus considerably overweighing errors made within ROIs with small macrophage numbers. Instead, we will use the Pearson correlation coefficients between the methods’ outputs for the complete sample of ROIs. Since the manual count as reference method gives no information about the area of the tagged cells, this measure has the further advantage to allow for an immediate comparison of count or area information without the necessity of a normalization of the latter.

For (S1) and (S5), we will further provide the percentage of manually counted macrophages which are *exactly matched* by the output of the respective method. Due to the reasons mentioned in the “Background” section, the relation between a detected feature and a manually tagged macrophage is to be considered as a matching not only in the case if the marking cross falls inside the convex hull of the detected feature. A matching is given nonetheless if the tag and the convex hull of the feature are mutually disjoint but visual inspection reveals that the convex hull covers the marked macrophage at least partly.

*c) Method comparison by means of the whole samples.* To the whole samples, methods (S1) − (S4) have been applied and subsequently compared. We provide first the Pearson correlation coefficients for the methods’ output for the CD14 ^+^/488 nm and CD163 ^+^/555 nm channels. Since, however, the evaluation subregions as well as the overall density of cells contained within them show considerable variation between the samples, the outputs will be appropriately normalized and then compared again. As normalizations for (S1), we calculate the *density*, which is given as total macrophage count divided by area of evaluation subregion, cf. Step 3 of Algorithm 1 above, as well as the *cell percentage*, which is given as total macrophage count diveded by estimated total number of cell nuclei within the evaluation subregion. The latter is obtained from the cartoon of the DAPI channel by masking all pixels with intensity less than 10 and dividing the number of the remaining pixels by 100. As normalizations for (S2) − (S4), we calculate the *area percentages*, which are given as cumulative macrophage area divided by the area of the corresponding evaluation subregion.

We consider a feature detected within the CD14 ^+^/ 488 nm channel as *double-stained* if at least 20 % of the area of its convex hull is covered by convex hulls of some features detected within corresponding CD163 ^+^/555 nm channel image. Note that the presence of a double staining does not influence the detection of a feature by methods (S1) − (S4) since the channels are analyzed independently from each other. However, the more completely and uniformly a given macrophage is stained, the more probable is the recognition of a possible double staining.

*d) Analysis of tumor subregions.* The tumor subregions have been analyzed with method (S1) only. Here, we will compare the full output with its 50 % and 25 % downsampling, considering only one half or one quarter of the tiles of the given tumor subregion dataset for evaluation. Further, we provide a comparison with the outputs of (S1) and (GE) for the corresponding whole tissue sample. The analysis is repeated with the normalized outputs of (S1), calculated as densities. All comparisons will be given in terms of Pearson correlation. Moreover, the percentage of double-stained features according to the above given definition will be recorded. Finally, we characterize the distribution of the feature sizes, which will be derived from the analysis of the CD14 ^+^/488 nm channel. Frequencies are obtained by counting up all features of a given size and subpopulation over the outputs for all 44 datasets.

### Comparison with gene expression data for the whole samples

Digital-multiplexed gene expression (DMGE) profiling was performed with the nCounter platform (NanoString, Seattle, OR, USA), targeting the genes of interest by digitally color-coded oligonucleotides. For a detailed description of the procedure, see [39, 40]. The data were further processed and normalized by the following three steps. First, we performed quality controls using the R package NanoStringQCPro [41]. Here, four samples were flagged and removed from subsequent analysis. Second, we added a pseudo count and normalized the data by dividing sample-wise through the geometric mean of the housekeeper genes (B2M, MTMR14, PGK1, ABCF1, EIF2B4, LDHA, CTCF, TBP, WDR55, POLR2B), and third, we multiplied the data with a factor of 1000 to bring them on a natural scale. We refer to the normalized gene expression values as to method (GE). Below, the normalized counts will be compared with the outputs of image morphometry in terms of Pearson correlation coefficients.

### Summary of methods’ application

In Tables 1 and 2, we provide a summary of the properties of the described macrophage counting approaches and the experiments performed with them. Note that, for the whole tissue samples, comparison of results of (S1) − (S4) is possible for 40 datasets, and of (S1) − (S4) and (GE) for 35 datasets while (S5) and (S6) have not been applied.

**Table 1:** Summary of segmentation methods’ properties

	(S1)	(S2)	(S3)	(S4)	(S5)	(S6)
Software type						
proprietary			∙	∙		
freeware extension of proprietary	∙	∙				
freeware					∙	∙
Input						
.png format	∙	∙	∙	∙	∙	∙
.ndpi(s) format			∙	∙		
Output						
count	∙				∙	
area		∙	∙	∙		∙
annotated image			∙	∙	∙	∙
feature mask	∙	∙			∙	∙
logfile	∙	∙				
Evaluation subregion						
prescribed					∙	∙
manual detection				∙		
automated detection	∙	∙	(∙)			
Threshold adaptation						
manually			∙	∙		
automated	∙	∙			n/a	n/a
Feature detection						
none			∙	∙		
rule-based	∙	∙				
by training set					∙	∙

Abbreviations: (MC) — manual count, (S1) — automated macrophage count from ROF filter based segmentation approach, (S2) — cumulative macrophage area from ROF filter based segmentation approach, (S3) — cumulative macrophage area from Tissue Studio software, (S4) — cumulative macrophage area from Halo software, (S5) — automated macrophage count from Mask R-CNN machine learning approach, (S6) — cumulative macrophage area from Mask R-CNN machine learning approach, (GE) — normalized gene expression values from nCounter platform

**Table 2:** Summary of methods’ application to image data

	Manual count	(S1)	(S2)	(S3)	(S4)	(S5)	(S6)	(GE)
ROIs								
# single-channel images at CD14 ^+^/488 nm analyzed	25	25	25	25	25	17	17	−
# single-channel images at CD163 ^+^/555 nm analyzed	25	25	25	25	25	18	18	−
DAPI channel used	no	no	no	no	no	no	no	−
Whole tissue samples								
# single-channel datasets at CD14 ^+^/488 nm analyzed	−	44	44	43	41	−	−	37
# single-channel datasets at CD163 ^+^/555 nm analyzed	−	44	44	43	41	−	−	37
DAPI channel used	−	yes	yes	yes	yes	−	−	no
Tumor subregions								
# single-channel datasets at CD14 ^+^/488 nm analyzed	−	44	−	−	−	−	−	−
# single-channel datasets at CD163 ^+^/555 nm analyzed	−	44	−	−	−	−	−	−
DAPI channel used	−	yes	−	−	−	−	−	−

## Results

### Application to ROIs

*a) Application of segmentation methods and its mutual correlation.* First, we present the results of the methods’ application to the ROIs. In Table 3, we describe the parameters of the outputs (minimal/maximal value, mean, median, standard deviation). Calculation comprises all 50 ROIs for (MC), (S1) − (S4) and a subset of 35 ROIs for (S5) − (S6) while the remaining 15 images have been used for the generation of training and validation data.

**Table 3:** Results of segmentation methods (ROIs)

Method	(MC)	(S1)	(S2)	(S3)	(S4)	(S5)	(S6)
Unit	#	#	*μ*m^2^	*μ*m^2^	*μ*m^2^	#	*μ* *m* ^2^
Min.	8	22	1780.0	499.0	210.8	9	1406.0
Max.	311	204	19999.4	55057.3	34920.9	60	9686.2
Mean	112.6	97.9	10083.2	17904.0	5291.5	33.7	5543.0
Median	75	78.5	8597.0	13608.0	2842.7	37	6014.4
St.dev.	83.3	47.2	4839.2	13420.7	6539.5	15.5	2568.9

Table 4 contains the survey of the Pearson correlation coefficients between manual count (MC) and output of methods (S1) − (S6). Again, the mutual correlations between (MC), (S1) − (S4) have been calculated on the base of the complete ROI dataset while correlations involving (S5) and (S6) are calculated on the subset of 35 ROIs where the outputs of the latter were available. Complete results of methods’ application to the ROIs are provided in Additional file 1.

**Table 4:** Correlation between segmentation methods (ROIs)

	(MC)	(S1)	(S2)	(S3)	(S4)	(S5)	(S6)
(MC)	−	0.9297	0.8944	0.9077	0.6201	0.2864	0.3195
(S1)	0.0000	−	0.9661	0.8901	0.6898	0.3533	0.3877
(S2)	0.0000	0.0000	−	0.8999	0.7050	0.4972	0.5282
(S3)	0.0000	0.0000	0.0000	−	0.7719	0.3369	0.3741
(S4)	0.0000	0.0000	0.0000	0.0000	−	0.3233	0.3532
(S5)	0.0953	0.0373	0.0024	0.0478	0.0581	−	0.9985
(S6)	0.0614	0.0214	0.0011	0.0268	0.0374	0.0000	−

*p*-values below the diagonal

We observe that the ROF filter based segmentation method (S1) shows the best correlation with the manual count (MC), namely 0.9297. This correlation is slightly better than (S3) and (S2) and clearly superior to (S4), (S5) and (S6). The relative order of the correlations between (S1) − (S4) is 0.9661:0.8901:0.6898.

*b) Exact matching of manually counted macrophages.* In Table 5, we provide the analysis of exact feature matchings between (MC) − (S1) resp. (MC) − (S5). Here, the total number of macrophages counted in (MC) is summed up over all 50 ROIs for the comparison with (S1) (column 2) and over the 35 ROIs available for analysis with (S5) (column 5).

**Table 5:** Exact matches between (MC) − (S1) and (MC) − (S5) (ROIs)

Method	(MC)	(S1)	(MC) − (S1)	(MC)	(S5)	(MC) − (S5)
	macrophages	features	exact matches	macrophages	features	exact matches
total number (#)	5632	4894	3724	4194	1180	1101
Percentages related to (MC)						
total (all 50 resp. 35 ROIs)	100.0	86.9	66.1	100.0	28.1	26.2
Percentages related to (S1) resp. (S5)						
total (all 50 resp. 35 ROIs)		100.0	76.1		100.0	93.3
Min.			20.8			33.3
Max.			97.5			100.0
Mean			71.0			91.7
Median			77.2			94.6
St.dev.			19.3			12.3

### Application to whole tissue samples

*a) Mutual correlation between segmentation methods.* For the application of (S1) − (S4) to the whole tissue samples, we compare first the obtained evaluation subregions in terms of Pearson correlation coefficients, see Table 6. For (S1), we include the estimated number of cell nuclei as well.

**Table 6:** Correlation of normalization bases (whole tissue samples)

	(S1)/(S2)	(S1)/(S2)	(S3)	(S4)
	eval. area	est. # nuclei	eval. area	eval. area
(S1)/(S2)	−	0.9418	0.9305	0.9314
eval. area				
(S1)/(S2)	0.0000	−	0.7930	0.7886
est. # nuclei				
(S3)	0.0000	0.0000	−	0.9974
(S4)	0.0000	0.0000	0.0000	−

*p*-values below the diagonal

In Table 7, we show the Pearson correlation coefficients between the outputs of methods (S1) − (S4) and the gene expression data (GE) for the CD14 ^+^/488 nm and CD163 ^+^/555 nm channels, respectively. In Table 8, we repeat the survey with the normalized outputs of (S1) − (S4). Calculations comprise 40 datasets for the mutual correlations between (S1) − (S4) and 35 datasets for correlations involving (GE).

**Table 7:** Methods’ correlation (whole tissue samples)

	CD14 ^+^/488 nm channel					CD163 ^+^/555 nm channel				
	(S1)	(S2)	(S3)	(S4)	(GE)	(S1)	(S2)	(S3)	(S4)	(GE)
(S1)	−	0.9909	0.7424	0.7181	0.3261	−	0.9803	0.8415	0.7675	0.6380
(S2)	0.0000	−	0.7367	0.7257	0.3020	0.0000	−	0.8589	0.7755	0.6257
(S3)	0.0000	0.0000	−	0.5959	0.2700	0.0000	0.0000	−	0.7288	0.7099
(S4)	0.0000	0.0000	0.0000	−	0.2545	0.0000	0.0000	0.0000	−	0.5821
(GE)	0.0559	0.0778	0.1168	0.1402	−	0.0000	0.0000	0.0000	0.0002	−

*p*-values below the diagonal

**Table 8:** Methods’ correlation (whole tissue samples), normalized outputs

	CD14 ^+^/488 nm channel						CD163 ^+^/555 nm channel					
	(S1)	(S1)	(S2)	(S3)	(S4)	(GE)	(S1)	(S1)	(S2)	(S3)	(S4)	(GE)
	density	cell perc.	area perc.	area perc.	area perc.		density	cell perc.	area perc.	area perc.	area perc.	
(S1) density	−	0.8150	0.9660	0.7972	0.4880	0.5961	−	0.8616	0.9606	0.7964	0.7765	0.7354
(S1) cell perc.	0.0000	−	0.7634	0.5809	0.4360	0.5543	0.0000	−	0.7873	0.6460	0.6732	0.6970
(S2)	0.0000	0.0000	−	0.8247	0.5772	0.6184	0.0000	0.0000	−	0.8548	0.8228	0.7924
(S3)	0.0000	0.0001	0.0000	−	0.4077	0.3139	0.0000	0.0000	0.0000	−	0.7612	0.6713
(S4)	0.0014	0.0049	0.0001	0.0090	−	0.3204	0.0000	0.0000	0.0000	0.0000	−	0.6384
(GE)	0.0002	0.0005	0.0001	0.0663	0.0606	−	0.0000	0.0000	0.0000	0.0000	0.0000	−

*p*-values below the diagonal

Macrophage densities, as observed by (S1) in all 44 datasets, range from 353.6 to 1374.6 cells/mm^2^ with a mean of 847.9±269.3 cells/mm^2^ for the CD14 ^+^/488 nm channel, and from 325.7 to 1715.4 cells/mm^2^ with a mean of 833.9±328.2 cells/mm^2^ for the CD163 ^+^/555 nm channel. Macrophage cell percentages resulting from (S1) range from 2.42 % to 11.29 % with a mean of 5.56±2.05 % for the CD14 ^+^/488 nm channel, and from 2.23 % to 10.87 % with a mean of 5.47±2.35 % for the CD163 ^+^/555 nm channel. Complete results of methods’ application to whole tissue samples are provided in Additional file 2.

The relative order of correlations between (S1) − (S4) is 0.9909:0.7424:0.7181 and 0.9803:0.8415:0.7675 in Table 7, and 0.9660:0.7972:0.4880 and 0.9606:0.7964:0.7765 in Table 8.

*b) Correlation with gene expression data.* In Tables 7 and 8, (GE) is correlated with the output of (S1) with coefficients of 0.3261, 0.6380, 0.5961 and 0.7354, respectively. For Table 7, column 4, this is the best value, while in Table 7, column 9, and Table 8, methods (S3), (S2) and (S2) are slightly better correlated with coefficients of 0.7099, 0.6184 and 0.7924, respectively. Otherwise, correlation between (GE) and the commercial software kits (S3) and (S4) is rather poor.

*c) Double-stained features.* In the output of (S1), we observed considerable numbers of double-stained features. Percentages range from 25.72 % to 77.68 % of the detected CD14-positive macrophages within a single dataset bearing CD163-positive staining as well. In the mean, 55.51 % of the macrophages per dataset detected by (S1) were double-stained.

### Application to tumor subregions

*a) Results of subsampling.* In Table 9, we show the Pearson correlation coefficients between the output of methods (S1) and (GE) for the whole tissue samples and the output of (S1) for the respective tumor subregions selected within them, subjected to 100 %, 50 % and 25 % sampling rate. In Table 10, we repeat the analysis with the macrophage densities instead of the counts. Calculations comprise 44 datasets for the mutual comparisons of (S1) and 37 datasets for the comparison with (GE). Note that the correlations between (S1) and (GE) in Tables 9 and 10 differ slightly from those in Tables 7 and 8 because of additional data involved in the calculation of the latter (37 instead of 35 datasets).

**Table 9:** Correlations under subsampling (whole tissue samples and tumor subregions)

	CD14 ^+^/488 nm channel					CD163 ^+^/555 nm channel				
	(S1)	(GE)	(S1)	(S1)	(S1)	(S1)	(GE)	(S1)	(S1)	(S1)
	whole	whole	TS	TS	TS	whole	whole	TS	TS	TS
			100 %	50 %	25 %			100 %	50 %	25 %
(S1), whole	−	0.3306	0.6550	0.6572	0.6535	−	0.6308	0.6749	0.6733	0.6735
(GE), whole	0.0457	−	0.4425	0.4434	0.4310	0.0000	−	0.5767	0.5745	0.5684
(S1), TS, 100 %	0.0000	0.0061	−	0.9994	0.9985	0.0000	0.0002	−	0.9996	0.9987
(S1), TS, 50 %	0.0000	0.0060	0.0000	−	0.9991	0.0000	0.0002	0.0000	−	0.9990
(S1), TS, 25 %	0.0000	0.0077	0.0000	0.0000	−	0.0000	0.0002	0.0000	0.0000	−

*p*-values below the diagonal

**Table 10:** Correlations under subsampling (whole tissue samples and tumor subregions), normalized outputs

	CD14 ^+^/488 nm channel					CD163 ^+^/555 nm channel				
	(S1)	(GE)	(S1)	(S1)	(S1)	(S1)	(GE)	(S1)	(S1)	(S1)
	whole	whole	TS	TS	TS	whole	whole	TS	TS	TS
			100 %	50 %	25 %			100 %	50 %	25 %
(S1), whole	−	0.6184	0.8528	0.8533	0.8555	−	0.7422	0.9148	0.9144	0.9113
(GE), whole	0.0000	−	0.7478	0.7444	0.7318	0.0000	−	0.8069	0.8068	0.8036
(S1), TS, 100 %	0.0000	0.0000	−	0.9991	0.9968	0.0000	0.0000	−	0.9994	0.9970
(S1), TS, 50 %	0.0000	0.0000	0.0000	−	0.9981	0.0000	0.0000	0.0000	−	0.9984
(S1), TS, 25 %	0.0000	0.0000	0.0000	0.0000	−	0.0000	0.0000	0.0000	0.0000	−

*p*-values below the diagonal

Macrophage densities, as observed by (S1) in all 44 fully evaluated datasets, range from 463.3 to 1574.9 cells/mm^2^ with a mean of 907.7±325.3 cells/ mm^2^ for the CD14 ^+^/488 nm channel, and from 371.3 to 1758.9 cells/mm^2^ with a mean of 836.9±376.9 cells /mm^2^ for the CD163 ^+^/555 nm channel. Macrophage cell percentages resulting from (S1) range from 2.17 % to 13.99 % with a mean of 5.93±2.62 % for the CD14 ^+^/488 nm channel, and from 1.98 % to 14.36 % with a mean of 5.46±2.82 % for the CD163 ^+^/555 nm channel.

Complete results of application of (S1) to tumor subregions are provided in Additional file 3.

*b) Double-stained features.* As to expect from our observations for the whole tissue samples above, double-stained features are fairly common in the output of (S1). Percentages range from 25.37 % to 75.95 % per fully evaluated dataset, with a mean percentage of 53.41 %.

**Fig. 5:**
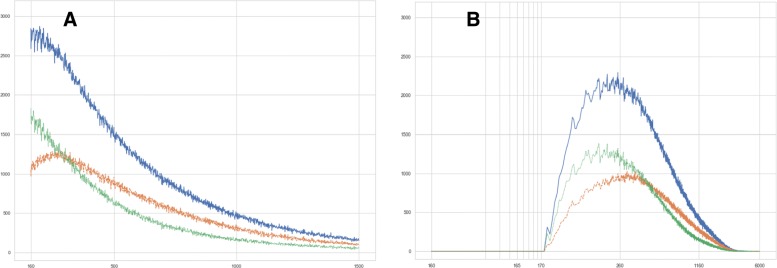
Histogram of feature sizes in tumor subregions, output of (S1), CD14 ^+^/488 nm channel. **a** — *x*-axis: size of detected features (px), linear scale. *y*-axis: sum of feature counts over all 44 analyzed datasets. Blue: all features, green: features without double staining, yellow: features bearing double staining. **b** — *x*-axis: size of convex hulls of detected features (px), logarithmic scale. *y*-axis: sum of feature counts over all 44 analyzed datasets. Colors as before

*c) Distribution of feature sizes.* Within the counts of features and convex hulls of features, we distinguish subpopulations with or without double staining. The properties of the obtained distributions (minimal/maximal value, mean, median, standard deviation, 95 % quantil) are summarized in Table 11. All feature sizes are given in px. The minimal feature sizes result from the choice of parameters *s*_*min*_=160 and *c*_*min*_=7.5, the maximal feature sizes in columns 2 − 4 reflect the setting of the parameter *s*_*max*_=1500. Figure 5 shows the histogram of the feature sizes.

**Table 11:** Distribution of feature sizes (px) in tumor subregions, output of (S1), CD14 ^+^/488 nm channel

Features	∙	∙	∙			
Convex hulls				∙	∙	∙
Staining resp. subpopulation	all	single	double	all	single	double
Min.	160	160	160	172	172	172
Max.	1500	1500	1500	6000	6000	5996
Mean	534.6	486.1	574.0	753.3	659.8	829.2
Median	445.5	390.5	494.5	570.5	482.5	654.5
St.dev.	313.6	298.5	320.0	549.8	501.2	575.2
95 % quantil	1195.5	1136.5	1231.5	1899	1736	1997

From Table 11, we observe a mean macrophage size of 152.5±111.3 *μ*m^2^. For the single-stained subpopulation, the mean size is 133.6±101.5 *μ*m^2^, slightly differing from the double-stained subpopulation with a mean size of 167.9±116.5 *μ*m^2^.

## Discussion

• Our results show that *the ROF filter based segmentation method (S1) may be considered as fairly reliable* and well-comparable with with other existing methods. Besides of showing the best correlation with the manual count (MC), the mean and median of (S1) and (MC) are closely related. Further, we see that the automated determination of evaluation subregions in (S1)/(S2) based on DAPI channel information is fully reliable. The relative order of correlations between (S1) − (S4) is comparable for the applications to ROIs and whole tissue samples. Our results further indicate that the different normalizations of (S1) (density and cell percentage) contain different information and must be indeed distinguished. As to expect, the percentage of exact matches between the features detected by (S1) and manually counted macrophages is lower than in situations where more regular shaped and uniformly stained cells are targeted. In view of the difficulties described in the “Background” section, the absolute and relative percentages of 66.1 % and 76.1 % of exactly matched macrophages, respectively, although moderately underestimating the absolute number of macrophages, are still fairly large. For large numbers of macrophages, cell counts by (S1) and area determination by (S2) turn out to be largely equivalent.

Of course, within the outputs of method (S1), one may observe the typical errors in automated cell counting, which would be avoided by a human examiner (cf. [25], p. 11, Fig. 4). While, on the one hand, tightly packed and uniformly stained macrophages may be lumped into a single feature, nonuniform staining of single macrophages may cause, on the other hand, a “breaking” of the cell image, resulting in a double or multiple count. For the same reason, many macrophages will be recognized only partly, thus be properly counted but inaccurately masked. The setting of the parameter *s*_*max*_ may exclude large single macrophages or aggregates of squeezed macrophages from counting. Background structures may be misidentified as macrophages as well.

**Fig. 6:**
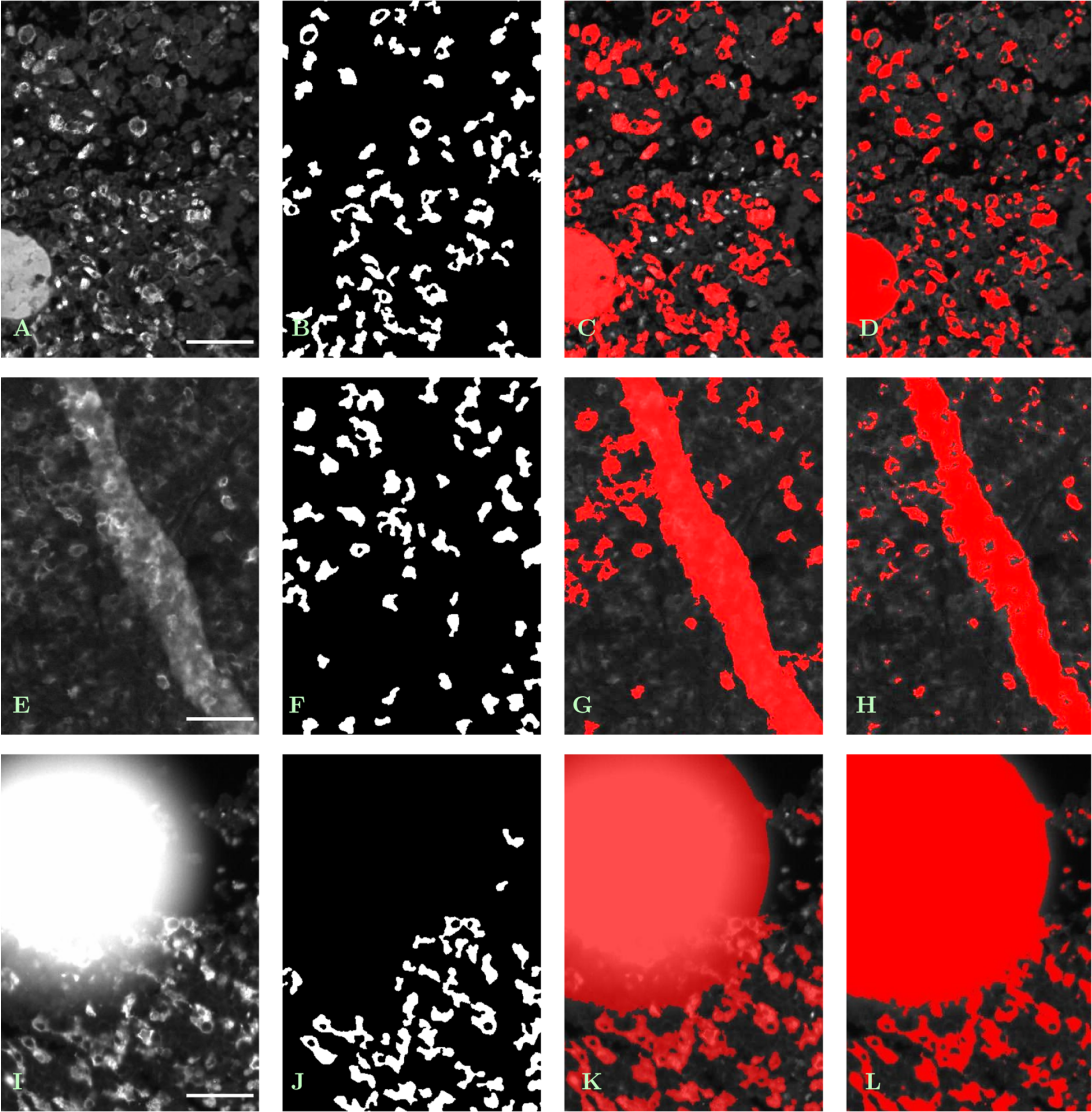
Segmentation methods (S1) - (S4) coping with problems in tissue preservation. Features have been identified based on information from all of three analyzed channels. **a** – **d***Sample with vessel (bottom left) and erythrocytes (middle).***a** — Original single-channel image (whole tissue sample No. 24, cutout from tile No. (42, 16), CD163 ^+^/555 nm), contrast enhanced by factor 1.5, scale bar 45 *μ*m. **b** — Result of (S1), (S2); vessel as a hyperfluorescent feature removed, erythrocytes partly ignored. **c** — Result of (S3), contrast enhanced by factor 1.5; vessel erroneously marked as target area, erythrocytes partly ignored. **d** — Result of (S4), contrast enhanced by factor 1.5; vessel as well as erythrocytes erroneously marked as target area. **e** – **h***Sample with tissue fold.***e** — Original single-channel image (whole tissue sample No. 31, cutout from tile No. (3, 20), CD14 ^+^/488 nm), contrast enhanced by factor 2, scale bar 45 *μ*m. **f** — Result of (S1), (S2); fold as a strongly fluorescent feature removed, macrophages under the fold partly detected. **g** — Result of (S3), contrast enhanced by factor 2; fold erroneously marked as target area. **h** — Result of (S4), contrast enhanced by factor 2; fold erroneously marked as target area. **i** – **l***Sample with staining artifact (splatter of staining liquid).***i** — Original single-channel image (whole tissue sample No. 11, cutout from tile No. (18, 21), CD163 ^+^/555 nm), scale bar 45 *μ*m. **j** — Result of (S1), (S2); splatter as a hyperfluorescent feature removed, macrophages close to its border properly detected. **k** — Result of (S3); splatter erroneously marked as target area. **l** — Result of (S4); splatter erroneously marked as target area

Nevertheless, method (S1) shows considerable robustness when dealing with scratches, folds, overstainings or splatters of staining liquid (which were excluded when selecting ROIs and tumor subsections but are present in the whole tissue samples). In Fig. 6, some typical examples are shown.

For the obtained cell counts, no stereological corrections [42] have been applied since the mean size of target macrophages largely exceeds the thickness of tissue slides.

• The *application of commercial software kits* to macrophage segmentation is confronted with serious difficulties. The above described selection of the analysis modes and parameters has been performed to the best of the authors’ experience. In particular, due to the heterogeneity of the data, the use of fixed thresholds turned out to be inappropriate. For the same reasons, we refrained from the application of cell counting modes with prior nucleus detection (based on synchronous DAPI staining of the samples) and subsequent colocalization of stained area around the nuclei. As a consequence, we must restrict ourselves to detection modes analyzing the stained area in single-channel fluorescence images, and the necessity of repeated manual interventions for parameter adaptation had to be accepted. Even under these preconditions, both software packages cope very poorly with artifacts in tissue preservation (typical examples are shown in Fig. 6). For the analysis of whole tissue samples Nos. 23 and 35, both suffering from overstaining and widespread presence of erythrocytes, application of (S4) (in the above described analysis mode) failed at all.

Let us further remark that our results reveal a considerable disagreement between the outputs of both commercial software kits with a correlation of 0.7719 for the ROIs and correlations ranging from 0.4077 to 0.7612 for the whole tissue samples.

Compared with the commercial software kits applied in this study, the ROF filter based segmentation method has the advantages of full automatization, complete documentation of the algorithm and exact repeatability. Tissue preservation artifacts are handled in a much more robust way. Moreover, shapes, sizes, positions and colocalization of macrophages can be observed from the method’s output.

• Straightforward application of the *Mask R-CNN machine learning approach* (S5)/(S6) leaded to very poor results in terms of correlation with (MC) as well as of the absolute percentage of exact matches between (MC) and (S5). The relative percentage of artifacts (6.7 %) generated by (S5), however, is considerably lower than in (S1). Nevertheless, although we used the common ratio of 20 %:10 %:70 % between training, validation and analysis data, it is obvious that the application of the neural network suffered from a strong deficiency of training items. As a consequence, we refrained from an application of (S5)/(S6) to whole tissue samples.

The window size for the training items has been selected in agreement with the mean macrophage area observed in Table 11.

• For the whole tissue samples as well as for the tumor subregions, *correlation coefficients for the CD163 staining are slightly larger than for CD14 staining* for all surveyed methods. This observation may be explained by the fact that the CD14 staining appears weaker than the CD163 staining. In general, such differences depend on the distribution of the epitop on the cell surface and the binding of the primary antibody. Experiments during the staining process revealed that the combination of the primary antibody CD14 with the fluorophore Alexa 488 resulted in the clearest possible images.

With regard to the possible nonuniformity of the staining of single macrophages, it is obvious that the *distribution of the macrophage sizes* should be observed from the convex hulls of the features rather than from the features themselves. The slighty increased mean size of the double-stained subpopulation may simply reflect the fact that the detection of a double staining is less probable for small cell fragments, dissected or cropped cells.

*Subsampling within the tumor subregions* leads to almost perfectly correlated results, which are mutually correlated with coefficients greater than 0.99. On the other hand, the discrepancies between the counts and densities obtained for the whole tissue samples and the tumor subregions cannot be neglected.

• In general, comparison between *image morphometry and gene expression analysis* reveals moderate to low correlation levels, regardless whether (GE) is compared with (S1)/(S2) or with the outputs of the commercial software kits (S3) and (S4). Further, we may observe that normalization of the outputs of (S1) − (S4) improves the correlations to a moderate level at best, and that correlations for the CD163 staining/expression are better than those for the CD14 staining/expression.

If tumor subregions are piloted instead of whole tissue samples, correlations shift in a nonuniform way without a considerable improvement.

• We may conclude that the ROF filter based segmentation method constitutes a *solid approach to obtain reliable counts and distributions for different macrophage types in IHC stained whole tissue samples*. Compared with counts of high power fields, the new method provides an easy access to a *complete* representation of the heterogeneous tumor microenvironment. In terms of Pearson correlation, results of gene expression profiling are not reproduced by morphometrical image analysis. In difference to GEP, ROF filter based segmentation is able to identify and to count double-labeled macrophages, thus enabling the study of diverse macrophage subpopulations. Moreover, the method allows for a systematic study of the local distribution of the macrophages, thus enabling subsequent investigations of macrophage clustering and applications of point pattern statistics.

As a future challenge, the detailed information about macrophage counts and distribution obtained by the ROF filter based segmentation method has to be tested for its prognostic potential in different lymphoma diseases. In a first step, we carried out a clinical application of the ROF method to a large cohort of DLBCL patients (*N*>400). Based on IHC stained TMAs, image data for the Alexa 488, Alexa 555 and DAPI channels were generated by the same protocol as described above. These images have been analyzed in full analogy to the tumor subsections, obtaining counts and densities for CD14- and CD163-positive macrophages, to be investigated for possible correlations with the documented clinical outcome. Again, we observed a fairly robust behaviour of the method, coping well with folds, scratches and overstainings in the tissue cores. Results will be reserved for a forthcoming publication.

## Conclusions

To the detection of IHC stained macrophages (CD14, CD163) in DLBCL tissue samples, a ROF filter based segmentation method has been successfully applied. The method, providing number, area, shape, and location of stained macrophages, is deterministic, fully automated, externally repeatable, independent on training data as well as on particular markers and completely documented. Comparison of macrophage counts obtained by ROF filter based segmentation with gene expression data reveals only moderate levels of correlation, thus indicating that image morphometry constitutes an independent source of information about antibody-polarized macrophage occurence and distribution.

## Additional files

Additional file 1 Analysis of ROIs. (XLS 30 kb)

Additional file 2 Analysis of whole tissue samples. (XLS 51 kb)

Additional file 3 Additional files 1, 2 and 3 contain the segmentation results for the ROIs, whole tissue samples and tumor subregions, respectively.
